# Longitudinal Associations Between ADHD Symptoms and Cannabis Use in Adolescence: Exploration of the Dynamic Cross-Lagged Effects

**DOI:** 10.1177/07067437261497468

**Published:** 2026-11-07

**Authors:** Julie Girard-Lapointe, Natalie Castellanos-Ryan, Samantha J. Lynch, Jeremy J. Watts, Maria Didaskalou, Patricia J. Conrod

**Affiliations:** 1 Centre de recherche Azrieli, CHU Sainte-Justine; 2 Département de psychiatrie et d'addictologie, Université de Montréal; 3 École de psychoéducation, Université de Montréal

**Keywords:** ADHD symptoms, cannabis use, adolescence, RI-CLPM, CoVenture

## Abstract

**Introduction:**

Adolescence represents a period of heightened vulnerability for the initiation of psychoactive substance use, such as cannabis. A growing body of research suggests a marked co-occurrence between attention-deficit/hyperactivity disorder (ADHD) symptoms and early or problematic cannabis use. However, most of these studies are cross-sectional, raising questions about the nature and directionality of this association. Understanding the underlying dynamics of this relationship is of particular importance for both prevention and intervention among at-risk adolescents.

**Method:**

Data were drawn from *CoVenture*, a longitudinal study of 3,756 secondary school students from the Greater Montreal area. Each year from Grade 7 to Grade 11, participants self-reported their frequency of alcohol and cannabis use, as well as ADHD symptoms. A Random Intercept Cross-Lagged Panel Model was used to assess within-person associations over time, controlling for alcohol use and demographic covariates.

**Results:**

At a between-person level, cannabis use and ADHD symptoms were significantly associated, even after controlling for alcohol use. At a within-person level, findings suggest a bidirectional relationship, starting in Grade 8. Greater ADHD symptom severity significantly predicted subsequent year increases in cannabis use. The reverse pathway—from greater cannabis use to subsequent year increases in ADHD symptoms—was also significant. Further analysis showed that higher cannabis use in Grade 9 increased the risk of reaching a clinically relevant level of ADHD symptoms in Grade 10.

**Conclusion:**

The findings suggest that ADHD and cannabis use are related through a common vulnerability mutually exacerbating relationship where (a) ADHD symptoms present a risk factor for cannabis use onset, and (b) once cannabis use has begun, cannabis use and ADHD symptoms appear to mutually predict each other, suggesting a potential “spiral effect” that warrants attention.

## Plain Language Summary

Do inattention/hyperactivity problems and cannabis use mutually worsen each other during adolescence ?

Why was the study done? Adolescence is a period of vulnerability to substances, such as cannabis. Many studies suggest that symptoms of attention deficit hyperactivity disorder (ADHD) and early cannabis use are linked. However, it is unclear whether it is simply that adolescents more at risk for one are more at risk for the other (between-person difference), or whether an increase in one then leads to an increase in the other (within-person difference). This is crucial, particularly for informing prevention and intervention strategies.

What did the researchers do? A total of 3,756 students were followed annually from grades 7 to 11. Participants self-reported their frequency of cannabis use and their ADHD symptoms. Analyses were carried out to verify both a) between-person differences and b) within-person changes over time.

What did the researchers find? Adolescents with a higher general level of ADHD symptoms also had a higher risk of using cannabis, compared to adolescents with lower ADHD symptoms. Regarding the within-person variations across time, higher frequency of cannabis use in one year was associated with an increase in ADHD symptoms the following year. Also, an increase in the severity of ADHD symptoms in one year was associated with a higher frequency of cannabis use the following year.

What do the findings mean? The findings suggest that not only a) ADHD and cannabis use are linked by a shared vulnerability, but also that b) cannabis use and ADHD symptoms mutually predict each other through time, suggesting a potential “spiral effect”. Adults supporting adolescents presenting high levels of ADHD symptoms should be mindful of their risk for early cannabis use, but also view cannabis use as a behavior that does exacerbate ADHD symptoms, and that therefore needs to be addressed.

## Résumé

**Introduction:**

Pour ce qui est de la consommation de substances psychoactives, comme le cannabis, l’adolescence représente une période de grande vulnérabilité. De plus en plus de recherches suggèrent une importante cooccurrence des symptômes du Trouble déficit de l’attention avec ou sans hyperactivité (TDAH) et de la consommation précoce ou problématique de cannabis. Cependant, il s’agit pour la plupart d’études transversales, ce qui soulève des questions sur la nature et la directionnalité d’une telle association. Comprendre la dynamique sous-jacente de cette relation est particulièrement important pour la prévention et l’intervention chez les adolescents à risque.

**Méthode:**

Les données proviennent de *CoVenture*, une étude longitudinale menée auprès de 3 756 étudiants du secondaire de la région du Grand Montréal. Chaque année de la 7^e^ à la 11^e^ année, les participants ont déclaré leur fréquence de consommation d’alcool et de cannabis, ainsi que leurs symptômes de TDAH. Un modèle croisé-décalé avec intercepts aléatoires (RI-CLPM) a été utilisé pour évaluer les associations intra-individuelles au fil du temps, avec ajustement pour la consommation d’alcool et des covariables démographiques.

**Résultats:**

Une forte association a été observée entre la consommation de cannabis et les symptômes de TDAH, même après l’ajustement pour la consommation d’alcool. Les données interindividuelles suggèrent une relation bidirectionnelle, à partir de la 8^e^ année. La présence de symptômes de TDAH plus sévères prédisait une augmentation subséquente de la fréquence de consommation de cannabis. La relation inverse – de la consommation plus élevée de cannabis vers les symptômes plus sévères de TDAH – était également significative. Une analyse additionnelle a montré qu’une consommation plus élevée de cannabis en 9^e^ année augmentait le risque d’atteindre un niveau cliniquement significatif de symptômes de TDAH en 10^e^ année.

**Conclusion:**

Les résultats suggèrent que le TDAH et la consommation de cannabis sont liés par une relation dans laquelle a) des symptômes de TDAH sont un facteur de risque de début de consommation de cannabis; b) la consommation de cannabis et les symptômes du TDAH semblent se renforcer mutuellement une fois la consommation de cannabis commencée, ce qui suggère un « effet de spirale » potentiel qui mérite une attention.

## Introduction

Attention-deficit/hyperactivity disorder (ADHD) is a neurodevelopmental disorder characterized by chronic difficulties with inattention and/or hyperactivity-impulsivity. It persists into adolescence in approximately 80% of cases and into adulthood in 50–65% of cases.^1^ ADHD symptoms have both (a) a stable trait-like component, on which the ADHD diagnostic criteria logic is based,^2^ and (b) a state-like component which, in both normative and clinical populations, somewhat fluctuates across time and context.^2^ Therefore, ADHD symptoms could be attenuated and/or exacerbated by biological, environmental and psychosocial factors. Namely, ADHD symptoms are influenced by certain situations, such as quality of parental practices, family or school climate, and lifestyle such as routine, sleep hygiene, and screen time.^3–5^

Adolescence is a critical developmental period marked by maturation of the prefrontal cortex and substantial changes in executive functions such as inhibition, planning, and cognitive flexibility,^6–8^ leading to more pronounced consequences of substance use, such as cannabis, compared to adulthood.^9,10^ Specifically, early cannabis use has been associated with later difficulties in sustained attention,^10–12^ working memory,^10,12^ short-term memory,^11–13^ planning and sequencing skills,^11^ processing speed, and response inhibition.^14^ Taken together, these findings suggest that early or regular cannabis use may cause or exacerbate symptoms such as inattention or impulsivity.^10,15^ However, meta-analyses and systematic reviews highlight that these studies are generally cross-sectional, retrospective, and do not measure baseline functioning and, therefore, do not allow for a clear understanding of the temporality or causality of the associations.^10,12^

Cannabis use is more prevalent among adolescents with ADHD than in the general population.^16–18^ Although about 17–29% of secondary school students report using cannabis in the past year,^19,20^ this proportion is estimated to double among those with ADHD.^21^ Similarly, individuals with ADHD have a two- to three-fold increased risk of developing a cannabis use disorder.^21,22^ Moreover, they tend to initiate cannabis use at younger ages and may develop dependence more rapidly.^18,22^ Between 38% and 60% of adolescents using cannabis meet ADHD diagnostic criteria, compared to an estimated prevalence of 6–10% in the general population.^23^

Several hypotheses have been proposed regarding the mechanisms linking ADHD symptoms to cannabis use. Because of their self-regulation difficulties, these adolescents are more likely to engage in behaviours without considering consequences and to make decisions aimed at immediate gratification,^24,25^ favouring choices that maximize instant reward at the expense of long-term goals. This translates into greater difficulty resisting temptations and increased likelihood of using cannabis when the opportunity arises.^24,25^ Additionally, some studies suggest that adolescents may use cannabis as an attempt to cope with ADHD symptoms and functional impairments.^26,27^ The proportion of individuals reporting cannabis use to achieve more stable functioning in life is higher among those with ADHD than among their peers without ADHD.^26^ Moreover, reduction or cessation of substance use following the initiation of appropriate pharmacological treatment for ADHD has been documented, supporting the self-medication hypothesis.^28,29^ However, findings are mixed about the benefits of pharmacological treatment to prevent future cannabis use.^30^

Overall, these findings raise the possibility that the relationship between cannabis use and ADHD symptoms may be bidirectional and mutually reinforcing. On the one hand, ADHD symptoms may lead to increased cannabis use; on the other hand, cannabis use may exacerbate cognitive symptoms such as inattention and impulsivity.^11,13,15^ High ADHD symptoms and substance use could also share a common vulnerability, with some individuals presenting a higher risk of both than others.^16,30^ It could also be a combination of both, supporting models such as the common underlying vulnerability-mutually exacerbating model proposed by Stewart and Conrod^31^ to explain the patterns of comorbidity between substance use disorders and some psychiatric conditions. However, most studies are cross-sectional, limiting the ability to verify causal or directional relationships. Furthermore, very few studies have examined reciprocal, dynamic associations between ADHD symptoms and cannabis use at the within-person level. Traditional approaches focus on between-person differences, which can introduce interpretational bias, i.e., wrongly assuming that the associations apply to within-person change in cannabis use and ADHD symptoms across time. Accounting for within-person variability is crucial, particularly during adolescence, to better understand developmental dynamics. Thus, although several studies have shown associations between ADHD symptoms and increased cannabis use,^32,33^ they do not allow for determining the direction of the effect and generally only capture between-person differences. Furthermore, considering the frequent co-occurring use of alcohol and cannabis,^34^ alcohol remaining the most frequent and earlier initiate substance among adolescent before cannabis,^35^ it is important that the effect of each be isolated.

## Current Study

This study aimed to examine the reciprocal longitudinal associations between cannabis use and symptoms of ADHD during adolescence, considering two levels of analysis:

1. Between-person level: Determine whether adolescents prone to high levels of ADHD symptoms are also at greater risk for cannabis use, beyond risk of alcohol consumption.
  - We hypothesised that: Average levels of cannabis use would be positively associated with average levels of ADHD symptoms across adolescence (Between-person level).
2. Within-person level: Assess whether within-person fluctuations in ADHD symptoms are associated with prospective variations (the following year) in cannabis use, and vice versa, beyond the effect of alcohol consumption.
  - We hypothesised that a bidirectional, mutually exacerbating relationship would be observed between ADHD symptoms and cannabis use throughout adolescence. More specifically,
    1. Within-person increases in cannabis use relative to an individual's own average would be associated with higher ADHD symptoms at the subsequent time point.
    2. Within-person increases in ADHD symptoms relative to an individual's own average would be associated with higher cannabis use at the subsequent time point.

## Method

### Sample

Data were drawn from CoVenture,^36^ a longitudinal population-based randomized controlled trial aimed originally at evaluating the effectiveness of PreVenture,^36,37^ a personality-targeted substance use prevention program. Descriptive statistics of the sample are presented in Table 1. The study included 3,756 adolescents from 31 secondary schools in the Greater Montreal area, followed annually from Grade 7 to Grade 11. Each year, participants were invited to complete a confidential web-based classroom survey focusing primarily on cognitive and behavioural functioning as well as psychoactive substance use.

**Table 1.** Descriptive Information of Sociodemographic Characteristics and Key Study Variables.

		Time 1	Time 2	Time 3	Time 4	Time 5
ADHD symptoms (mean/SD)		3.9 (2.3)	4.1 (2.4)	4.1 (2.4)	4.1 (2.4)	4.1 (2.4)
ADHD symptoms (%)						
	Under clinical cutoff	86.6	84.4	83.7	82.7	84.5
	Above clinical cutoff	13.4	15.6	16.3	17.3	15.5
Age (mean/SD)		12.8 (0.5)				
Alcohol use (mean/SD)		0.4 (0.7)	0.7 (0.7)	0.9 (0.9)	0.5 (1.1)	0.7 (1.2)
Cannabis use (mean/SD)		0.1 (0.5)	0.2 (0.6)	0.4 (0.9)	1.2 (1.0)	1.5 (1.0)
Tobacco (mean/SD)		0.1 (0.5)	0.1 (0.6)	0.3 (0.8)	0.4 (1.0)	0.4 (1.0)
Condition (%)						
	Intervention	57.7				
	Control	42.3				
Conduct problem (%)						
	Under clinical cutoff	91.0	92.0	90.7	91.8	93.8
	Above clinical cutoff	9.0	8.0	9.3	8.2	6.2
Emotional problem (%)						
	Under clinical cutoff	93.7	92.9	90.8	90.3	89.6
	Above clinical cutoff	6.3	7.1	9.2	9.7	10.4
Generation status (immigration)						
	3rd generation or more	55.2%				
	2nd generation	32.3%				
	1st generation	11.9%				
	Unknown	0.6%				
Region of birth (%)						
	Canada or United States	87.5				
	Europe	2.4				
	Africa	1.7				
	East or South Asia	3.3				
	Middle East	0.9				
	South or Central America	2.6				
	Other/don’t know	1.6				
Sex (%)						
	Male	50.6				
	Female	49.4				
School's language (%)						
	French	65.7				
	English	34.3				
Tobacco (mean/SD)		0.1 (0.5)	0.1 (0.6)	0.3 (0.8)	0.4 (1.0)	0.4 (1.0)

*Note:* ADHD = attention-deficit/hyperactivity disorder.

The majority of students attended French-language schools (67.5%) and were born in Canada/United States (87.5%), which is consistent with population norms for the Island of Montreal during the same period.^38^ Most of the sample was not presenting with problematic substance use according to screening guidelines (95%)^39^ nor reporting a clinical level of mental health global difficulties (94.2%)^40^ at baseline. Clinical level of self-reported ADHD symptoms was present in 13.4% of the sample. Clinical level of self-reported emotional problem and conduct problem were respectively present in 6.3% and 9.0% of the sample. At baseline, 36.9% of the sample reported alcohol use, and 4.7% reported cannabis use.

Ethical approval was obtained from the Research Ethics Board of CHU Sainte-Justine in Montreal. All students provided active assent to participate, with parents also confirming their approval through written consent when required according to school procedure. To ensure confidentiality and maximize response reliability, participants were informed that neither parents nor teachers would have access to their responses, and that responses would be automatically anonymized.

Participants were excluded from the CoVenture Trial if at baseline they: (a) reported using a sham drug, or (b) exhibited unusual response patterns (e.g., identical answers across items).^37^

### Main variables

#### ADHD Symptoms

Adolescents completed the *Strengths and Difficulties Questionnaire* (SDQ) at all five measurement points. They reported their symptoms on a scale ranging from 0 (*not true*) to 2 (*certainly true*) at each wave. The *hyperactivity/inattention* subscale consists of five items (α = .70 to .77 across time points). The SDQ has been validated for use in clinical practice as well as in community and epidemiological settings across various countries.^41^ Previous studies have demonstrated good psychometric properties, including adequate convergent validity with other ADHD measures and a stable factor structure.^40^

#### Cannabis Use

Each school year, participants completed the *DEP-ADO* (*Dépistage de la consommation problématique d’alcool et de drogues chez les adolescents*),^39^^,^^42^ reporting their frequency of cannabis use on a scale ranging from 0 (*never*) to 5 (*daily*). The DEP-ADO is widely used in Quebec schools for screening substance use habits and identifying risk for problematic use. It demonstrates strong psychometric properties, including adequate internal consistency (α = .84), good convergent validity with other substance use measures,^39^ and discriminant validity for identifying youth at risk of developing a substance use disorder.^39,43^

### Control Variables

Control variables were selected based on previous research with the present sample and literature on characteristics potentially associated with ADHD symptoms and cannabis use.^5,44^ Namely, lower socioeconomic status (SES) and being a boy are associated with both higher risk for cannabis use and ADHD symptoms.^35,45,46^ Younger adolescents tend to present higher ADHD symptoms yet lower cannabis use.^35,47^

#### Age

Age was calculated based on the difference between the date the questionnaire was completed and the birth month and year reported by participants at baseline.

#### Socioeconomic Status

The *Family Affluence Scale* (FAS) was used to estimate socioeconomic status based on five indicators: amount of pocket money, number of family-owned cars, whether the adolescent has their own bedroom, family vacations, and personal computer ownership.^48^ This tool is commonly used to assess SES among children and adolescents through simple self-reported data, avoiding reliance on standard questions such as parental income and education level, which adolescents often have limited or imprecise knowledge of.

#### Sex

Sex was self-reported at baseline in response to the question: “*Are you a boy or a girl?*.”

#### Alcohol Use

Each school year, participants reported their frequency of alcohol consumption using the DEP-ADO (described above), on a scale ranging from 0 (*never*) to 5 (*daily*).

#### School

An anonymized number corresponding to the school attended was available for each participant, so that this number was common to all participants attending the same school and different from students attending another school. The school was used for school-level clustering.

#### Intervention Status

Participants were categorized as being either in the intervention or the control group.

## Analytical Approach

To examine the bidirectional longitudinal associations between cannabis use and ADHD symptoms, a Random Intercept Cross-Lagged Panel Model (RI-CLPM) was employed using Mplus version 8.8. This approach allows for a more precise estimation of within-person change over time by disentangling stable between-person differences from time-varying within-person fluctuations^49^ (Figure 1A).

**Figure 1.**
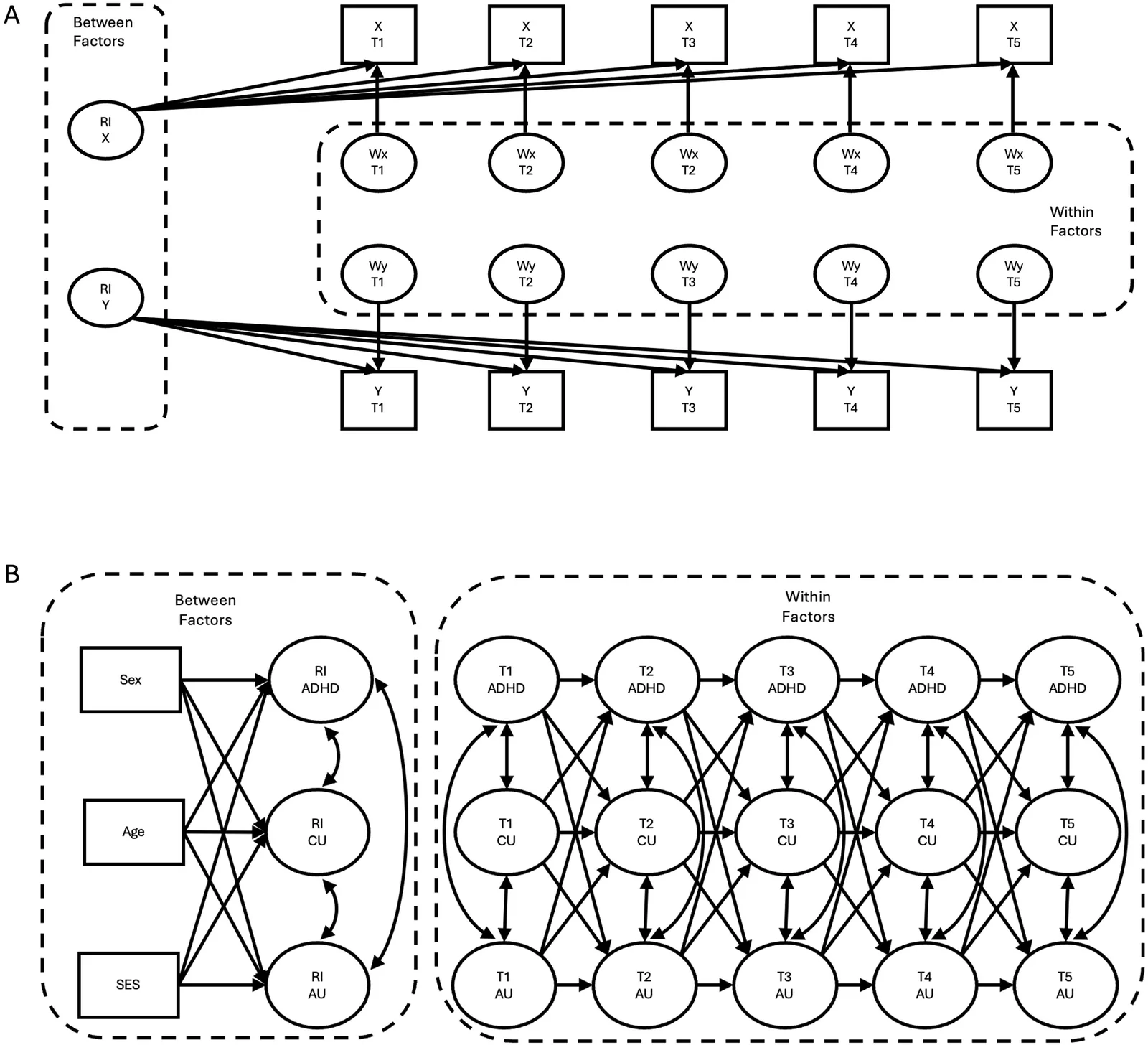
Visual representation of an RI-CLPM. (A) Conceptual modelling of an RI-CLPM. (B) Statistical model of the relationship between ADHD symptoms and cannabis use across secondary school, accounting for significant covariates.

RI-CLPM = Random Intercept Cross-Lagged Panel Model; ADHD = attention-deficit/hyperactivity disorder.

As shown in Figure 1B, random intercepts were specified for the two main constructs (cannabis use and ADHD symptoms) as well as for alcohol use. At the within-person level, we estimated autoregressive and cross-lagged effects across adjacent time points between ADHD symptoms, cannabis use and alcohol use. Residual covariances were included between constructs within the same time point. Random intercepts were allowed to covary, capturing stable associations among constructs. Random intercepts were also regressed on additional covariates (age, sex, and SES). Intervention status was not included in final models, based on results showing no added value or changes in overall results when intervention status was included as a covariate, and in line with previous research with this sample.^44^ To account for the hierarchical structure of the data (students nested within schools), models were estimated with a cluster correction for schools. Indirect effects were evaluated using the Mplus non-parametric bootstrap procedure with 5,000 sampling.

To improve the clinical relevance of our analyses, a second model using the presence of a significant level of ADHD symptoms instead of the dimensional ADHD symptoms variable was then carried out. We applied the widely used cutoff of seven on the SDQ subscale to identify youths presenting with a clinically significant level of ADHD symptoms.^50^ To confirm the similarity of the effects between boys and girls, a third model, stratified by sex (boy/girl), was also performed and is presented as Supplemental Table S1.

Model fit was evaluated based on conventional cutoffs (CFI and TLI ≥ .95, RMSEA ≤ .06, SRMR ≤ .08, χ^2^/df < 3).^51^ Effect sizes were evaluated based on benchmark values recommended for these models (.03 small, .07 medium, .12 large).^52^

## Results

### Descriptives and Correlations

Means, standard deviations and frequencies are shown in Table 1. Correlations among study variables are shown in Table 2. As expected, (a) within-variable correlations across time and (b) correlations between ADHD symptoms and substance use at each measurement point were significant.

**Table 2.** Correlations Among Study Variables.

	Correlations (*r*)																	
	1	2	3	4	5	6	7	8	9	10	11	12	13	14	15	16	17	18
1. Age T1	1																	
2. Sex T1	−.00	1																
3. SES T1	.03	.02	1															
4. Alcohol T1	.16***	−.10***	.12***	1														
5. Alcohol T2	.14***	−.01	.13***	.52***	1													
6. Alcohol T3	.11***	.03	.15***	.43***	.59***	1												
7. Alcohol T4	.09***	−.03	.14***	.34***	.48***	.62***	1											
8. Alcohol T5	.07***	−.05*	.15***	.33***	.44***	.52***	.63***	1										
9. Hash T1	.20***	−.07***	−.01	.33***	.18***	.15***	.11***	.14***	1									
10. Hash T2	.14***	.03	.06***	.26***	.32***	.23***	.22***	.18***	.38***	1								
11. Hash T3	.10***	.00	.04*	.27***	.31***	.44***	.34***	.29***	.27***	.54***	1							
12. Hash T4	.10***	−.05**	.04*	.27***	.33***	.42***	.47***	.37***	.30***	.43***	.64***	1				.		
13. Hash T5	.07***	−.09***	.05**	.25***	.31***	.40***	.44***	.47***	.26***	.32***	.50***	.70***	1					
14. ADHD T1	.04**	−.00	.00	.15***	.18***	.16***	.15***	.09***	.12***	.14***	.16***	.18***	.18***	1				
15. ADHD T2	.04*	.04*	.03	.12***	.19***	.17***	.14***	.11***	.07***	.16***	.17***	.17***	.18***	.61***	1			
16. ADHD T3	.01	.05**	.01	.10***	.16***	.19***	.16***	.12***	.05**	.13***	.20***	.19***	.22***	.54***	.66***	1		
17. ADHD T4	.01	.03	.02	.09***	.12***	.12***	.14***	.10***	.07***	.13***	.15***	.19***	.19***	.50***	.59***	.69***	1	
18. ADHD T5	−.00	.01	.02	.07***	.11***	.12***	.12***	.11***	.06**	.09***	.13***	.14***	.19***	.48***	.53***	.62***	.71***	1

*Note:* SES = Socioeconomic Status; ADHD = attention-deficit/hyperactivity disorder.

Sex: boy = 1, girl = 2.

*p-value ≤ .05; **p-value ≤ .01; ***p-value ≤ .001.

### Main Analysis

Results of the RI-CLPM are presented in Table 3 and summarize in Figure 2. Indicators show good fit for the model (Supplemental Table S2).

**Figure 2.**
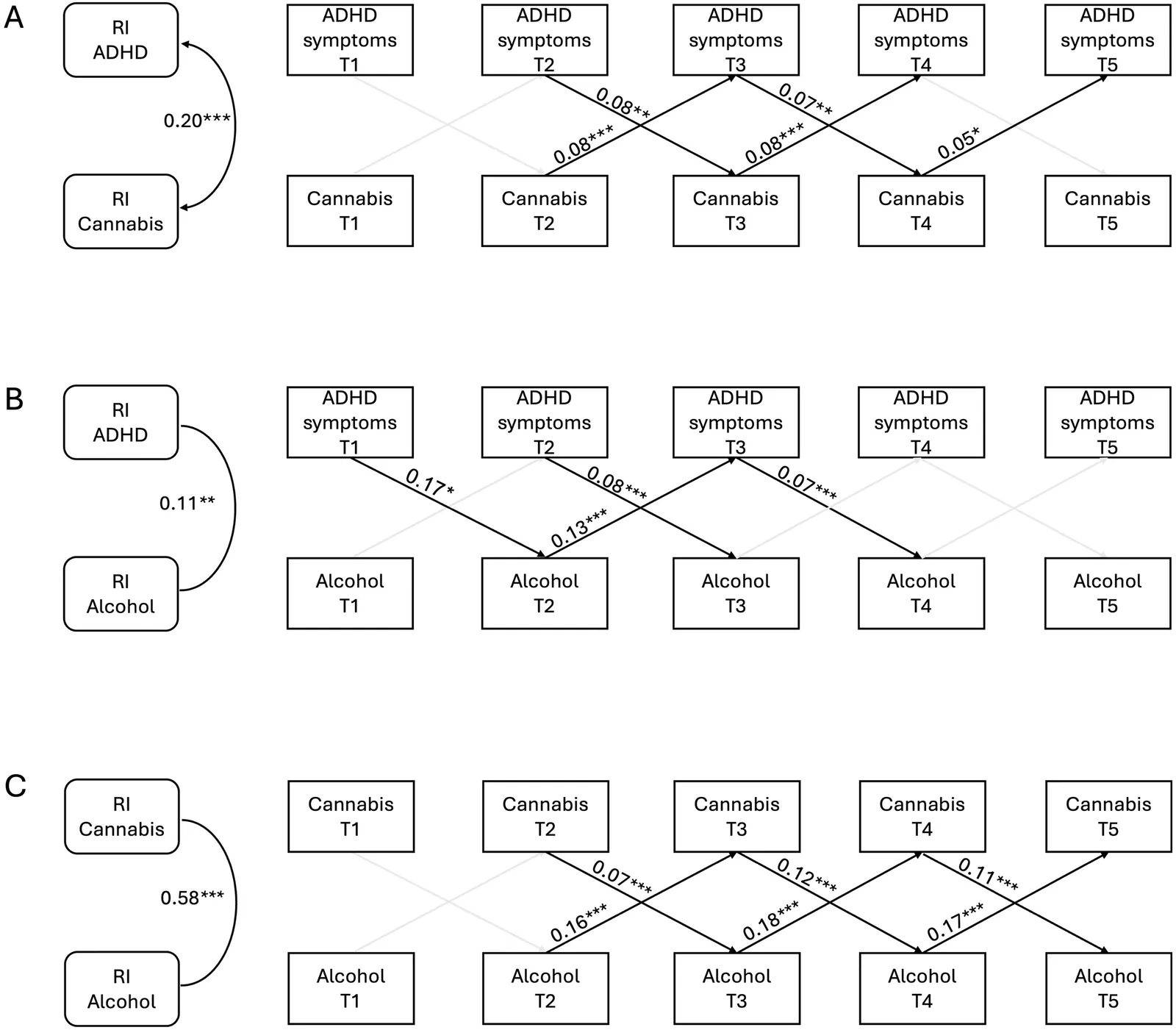
Summary of between- and within-person effects for Model 1. (A) ADHD symptoms and cannabis use. (B) ADHD symptoms and alcohol use. (C) Cannabis use and alcohol use. *Note*. Although integrated within the same model, the paths are presented here in variable pairs to facilitate reading.

**Table 3.** Path Coefficients for the RI-CLPM.

		T1		T2		T3		T4		T5	
		β	SE	β	SE	β	SE	β	SE	β	SE
**Model 1**	
	**Random intercepts**										
	RI by ADHD_t_	0.701***	0.015	0.700***	0.014	0.685***	0.014	0.686***	0.014	0.687***	0.013
	RI by cannabis_t_	0.885***	0.046	0.630***	0.064	0.444***	0.056	0.391***	0.051	0.346***	0.047
	RI by alcohol_t_	0.647***	0.040	0.568***	0.037	0.494***	0.034	0.439***	0.029	0.422***	0.031
	**Within factor**										
	ADHD	0.713***	0.014	0.714***	0.014	0.728***	0.013	0.728***	0.013	0.727***	0.012
	Cannabis	0.465***	0.103	0.777***	0.053	0.896***	0.028	0.920***	0.022	0.938***	0.017
	Alcohol	0.763***	0.034	0.823***	0.026	0.870***	0.019	0.899***	0.014	0.907***	0.015
	**Covariates**										
	Age → RI ADHD	0.043t	0.025								
	Age → RI cannabis	0.251***	0.057								
	Age → RI alcohol	0.241***	0.055								
	Sex → RI ADHD	0.015	0.027								
	Sex → RI cannabis	−0.067**	0.023								
	Sex → RI alcohol	−0.095**	0.032								
	SES → RI ADHD	0.006	0.021								
	SES → RI cannabis	0.003	0.027								
	SES → RI alcohol	0.204***	0.035								
	RI ADHD → RI cannabis	0.199***	0.034								
	RI ADHD → RI alcohol	0.111**	0.038								
	RI cannabis → RI alcohol	0.582***	0.063								
	**Within-person effects**										
	ADHD_t_ → ADHD_t+1_	0.225***	0.045	0.296***	0.039	0.377***	0.027	0.436***	0.021		
	Cannabis_t_ → cannabis_t+1_	−0.079	0.899	0.424***	0.042	0.509**	0.046	0.584**	0.028		
	Alcohol_t_ → alcohol_t+1_	0.240	0.423	0.430***	0.033	0.477***	0.023	0.509***	0.021		
	ADHD_t_ → cannabis_t+1_	0.095	0.156	0.076**	0.025	0.071**	0.023	0.045 ***^+^***	0.024		
	ADHD_t_ → alcohol_t+1_	0.165*	0.085	0.078***	0.023	0.073***	0.023	0.024	0.030		
	Cannabis_t_ → ADHD_t+1_	−0.116	0.186	0.081***	0.025	0.075***	0.022	0.045*	0.020		
	Cannabis_t_ → alcohol_t+1_	−0.158	0.473	0.065***	0.019	0.116***	0.031	0.110***	0.026		
	Alcohol_t_ → ADHD_t+1_	0.077	0.169	0.128***	0.028	0.012	0.026	0.038 ***^+^***	0.021		
	Alcohol_t_ → cannabis_t+1_	0.075	0.773	0.162***	0.027	0.183***	0.038	0.174***	0.019		
	**Residual covariances**										
	ADHD_t_ ↔ cannabis_t_	−0.016	0.066	0.103	0.106	0.135***	0.018	0.073***	0.022	0.088***	0.025
	ADHD_t_ ↔ alcohol_t_	0.178***	0.025	0.160	0.227	0.115***	0.028	0.043 ***^+^***	0.025	0.059**	0.023
	Cannabis_t_ ↔ alcohol_t_	−0.074	0.192	0.169	0.327	0.339***	0.023	0.282***	0.019	0.293***	0.025
**Model 2**	
	**Random intercepts**										
	RI by ADHD_t_	0.544***	0.022	0.521***	0.018	0.510***	0.020	0.495***	0.018	0.515***	0.015
	RI by cannabis_t_	0.870***	0.056	0.617***	0.069	0.435***	0.058	0.385***	0.053	0.340***	0.048
	RI by alcohol_t_	0.654***	0.040	0.573***	0.038	0.498***	0.034	0.442***	0.029	0.426***	0.031
	**Within factor**										
	ADHD	0.839***	0.014	0.853***	0.011	0.860***	0.012	0.869***	0.010	0.857***	0.009
	Cannabis	0.494***	0.125	0.787***	0.058	0.900***	0.029	0.923***	0.023	0.940***	0.018
	Alcohol	0.756***	0.035	0.819***	0.026	0.867***	0.019	0.897***	0.014	0.904***	0.015
	**Covariates**										
	Age → RI ADHD	0.023	0.026								
	Age → RI cannabis	0.256	0.056								
	Age → RI alcohol	0.241***	0.053								
	Sex → RI ADHD	0.046*	0.022								
	Sex → RI cannabis	−0.069**	0.024								
	Sex → RI alcohol	−0.097**	0.032								
	SES → RI ADHD	0.016	0.018								
	SES → RI cannabis	0.002	0.027								
	SES → RI alcohol	0.202***	0.034								
	RI ADHD → RI cannabis	0.181***	0.049								
	RI ADHD → RI alcohol	0.104*	0.054								
	RI cannabis → RI alcohol	0.599***	0.062								
	**Within-person effects**										
	ADHD_t_ → ADHD_t+1_	0.118**	0.044	0.247***	0.041	0.305***	0.036	0.270***	0.041		
	Cannabis_t_ → cannabis_t+1_	0.041	1.252	0.440***	0.044	0.522***	0.046	0.591***	0.027		
	Alcohol_t_ → alcohol_t+1_	0.247	0.622	0.445***	0.033	0.484***	0.024	0.512***	0.022		
	ADHD_t_ → cannabis_t+1_	0.060	0.183	0.022	0.028	0.035	0.021	0.009	0.018		
	ADHD_t_ → alcohol_t+1_	0.099	0.107	0.014	0.024	0.052 ***^+^***	0.029	−0.008	0.024		
	Cannabis_t_ → ADHD_t+1_	−0.066	0.237	0.049 ***^+^***	0.028	0.063*	0.032	0.029	0.027		
	Cannabis_t_ → alcohol_t+1_	−0.153	0.693	0.074***	0.020	0.122***	0.030	0.112***	0.026		
	Alcohol_t_ → ADHD_t+1_	0.042	0.207	0.102***	0.026	0.003	0.025	0.067*	0.030		
	Alcohol_t_ → cannabis_t+1_	0.083	1.060	0.174***	0.026	0.183***	0.039	0.175***	0.020		
	**Residual covariances**										
	ADHD_t_ ↔ cannabis_t_	0.022	0.069	0.075	0.192	0.092***	0.024	0.041 ***^+^***	0.023	0.066*	0.026
	ADHD_t_ ↔ alcohol_t_	0.120***	0.030	0.091	0.142	0.083***	0.021	0.035	0.023	0.055*	0.023
	Cannabis_t_ ↔ alcohol_t_	−0.087	0.230	0.188	0.924	0.341***	0.023	0.284***	0.018	0.294***	0.025

*Note*: SES = socioeconomic status; ADHD = attention-deficit/hyperactivity disorder; β *= standardized beta; SE* *=* *standard error.*

***^+^*** *p-value*  ≤ .10; *p-value  ≤ .05; **p-value ≤ .01; ***p-value ≤ *.001*.

Model fits. Model 1 (Intensity of ADHD symptoms): χ^2^(87) *= *336.695, *p* = .00, CFI = .988, TLI = .979, RMSEA = .028, 90%Cl [.024, .031], SRMR *= *.034. Model 2 (ADHD clinical cut-off): χ^2^(87) *= *324.671, *p* = .00, CFI = .982, TLI = .968, RMSEA = .027, 90%Cl [.024, .030], SRMR *= *.034.

For ADHD symptom severity, random intercept loadings were high and relatively stable across time points (λ = .69 to .70), indicating that a substantial portion of the variance in ADHD symptoms was explained by a stable between-person component. Similarly, loadings on the time-specific latent factors were also high (λ = .71 to .73), suggesting that individual-specific temporal variance was well captured by the model.

For cannabis use, random intercept loadings decreased progressively over time, from .89 at T1 to .35 at T5. This decrease suggests that shared variance with the stable between-person component was stronger in early adolescence and diminished over time. In contrast, loadings for the within-person components, reflecting personal fluctuations around the individual mean, increased substantially over time (λ = .47 at T1 to .94 at T5), indicating an increase in within-person variance across adolescence.

At the between-person level, the association between cannabis use and ADHD symptoms was significant (*r* = 0.20, *p* < .001), suggesting that greater overall cannabis use was associated with greater overall severity of ADHD symptoms.

Within-person cross-lagged paths indicated that further increases in ADHD symptoms (over and above the individual's intercept) predicted an increase in cannabis use at the next time point between Grade 8 and Grade 10 (T2 → T3: β = 0.08, *p* < .01; T3 → T4: β = 0.07, *p* < .01; T4 → T5: β = 0.05, *p* = .06). Conversely, increases in cannabis use predicted an increase in ADHD symptoms at the subsequent time point between Grade 8 and Grade 11 (T2 → T3: β = 0.08, *p* < .001; T3 → T4: β = 0.08, *p* < .001; T4 → T5: β = 0.05, *p* < .05). Effect sizes for these cross-lagged paths from ADHD to cannabis use were medium to large from T2 to T4, and small to medium from T4 to T5. Effect sizes for cross-lagged paths from cannabis to ADHD were also medium to large from T2 to T4, and small to medium from T4 to T5.

Bootstrap-based test of indirect effects for these cross-lagged paths (Supplemental Table S3) showed a significant indirect effect from cannabis use at T2 to cannabis use at T4 through ADHD symptoms at T3 (β = 0.01, 95% CI [.002, .014]).

Residual correlations between the within-person components of ADHD symptoms and cannabis use were nonsignificant at the first and second waves, but became positive and significant from the third wave onward (T3: *r* = 0.14, *p* < .001; T4: *r* = 0.07, *p* < .001; T5: *r* = 0.09, *p* < .001).

Additional analysis (Model 2, Table 3) using a clinically significant level of ADHD symptoms showed that at the between-person level, the association between cannabis use (overall level of use) and overall clinical level of ADHD symptoms was significant (*r* = 0.18, *p* < .001). At the within-person level, greater cannabis use in Grade 8 and Grade 9 predicted increases in ADHD symptoms to a clinically relevant level at the subsequent time point (T2 → T3: β = 0.05, *p* < .10; T3 → T4: β = 0.06, *p* < .05). In contrast, clinical level of ADHD symptoms at a given timepoint did not significantly predict an increase in cannabis use at the subsequent time point.

The sex-stratified analyses (Supplemental Table S1) confirm that the bidirectional relationship between cannabis use and ADHD symptoms is present in both boys and girls.

## Discussion

The present study aimed to examine the reciprocal longitudinal associations between cannabis use and ADHD symptoms during adolescence, while distinguishing within-person effects from stable between-person differences. To our knowledge, this is one of the first studies to test the directionality of the relationship between cannabis use and ADHD symptoms by (a) separating within-person fluctuations from between-person traits and (b) accounting for longitudinal changes throughout adolescence.

At the within-person level, our findings are consistent with a bidirectional relationship between ADHD symptoms and cannabis use. The effects of ADHD symptoms on future cannabis use appear in Grade 8 and subside in later adolescence, whereas the effects of cannabis use on future ADHD symptoms also begin to be observable in Grade 8 and remain significant throughout adolescence. In contrast, the effects of alcohol use on future ADHD symptoms were less consistent and limited to one time point.

Taken together, these findings support a common vulnerability-mutually exacerbating hypothesis, in which (a) ADHD symptoms present a risk factor for cannabis use onset, and (b) increases in ADHD symptoms and increases in cannabis use (above a person's own mean level) amplified each other in adolescence. Generally, the results underscore both the importance of monitoring ADHD symptoms to prevent the development and exacerbation of risky behaviours, namely substance use, and the importance of targeting cannabis use to prevent worsening of ADHD symptoms.

Controlling for alcohol use also allows us to distinguish the effect of cannabis from other substances. On one hand, our findings confirm that a higher overall level of ADHD symptoms is associated with a greater risk of cannabis use during adolescence, as well as more general substance use. On the other hand, the observed increase in ADHD symptoms following higher cannabis use in the previous year cannot simply be explained by polysubstance use or a common vulnerability to substance use. The specificity or extent of this effect, including the examination of potential sex-differentiated variations, should be further explored from a neuropharmacological perspective, as there is mounting evidence of a negative effect of early cannabis use on brain maturation.^43^

### Strengths and Limitations

This study contributes to the literature by clearly disambiguating longitudinal associations between cannabis use and ADHD symptoms at both between- and within-person levels. To our knowledge, this is one of the first studies to do so. Two major strengths include the large sample size and the five-year longitudinal follow-up, which enabled the implementation of modelling using multi-level random-intercept cross-lagged panel models. While effect sizes were modest, they help disentangle the directionality of these associations. Overall, our findings support a bidirectional relationship, in which cannabis use and ADHD symptoms are mutually exacerbating.

However, some limitations should be noted. First, the measures used have inherent constraints. The cannabis use measure did not capture quantity or potency, which could influence results. Additionally, the absence of information on ADHD diagnoses and medication limits the generalizability of findings to youth with clinically diagnosed ADHD. Identifying clinically significant symptom levels does not guarantee that full diagnostic criteria (e.g., chronicity, functional impairment) are met, and the SDQ scale cutoff has been shown to lack sensitivity for youths with ADHD who are not presenting the combination of high levels of inattention with high levels of hyperactivity/impulsivity.^50^ Moreover, youth with an established ADHD diagnosis but receiving pharmacological treatment may not report clinically significant levels of ADHD symptoms, even though they have the disorder. Additionally, while reliance on self-report data provides useful insights, it can also introduce further limitations. Overall, research supports the validity of self-reported substance use.^53,54^ However, findings for self-reported ADHD symptoms are less consistent. Some studies support the validity,^55,56^ while other studies suggest that youth with the highest ADHD symptom levels are often the least accurate informants^57,58^ and that adolescents tend to under-report their ADHD symptoms compared to their parents and teachers.^59^ Therefore, incorporating additional informants (e.g., parents, teachers) could have strengthened the assessment. Also, the sample overall reported low substance use. While this limits the detection of stronger effects, our findings still suggest meaningful associations between cannabis use and ADHD symptoms, even in a low-use sample. Finally, the data of the present study was collected between 2012 and 2018, the pre-COVID period and pre-legalization of cannabis in Canada. Thus, it would be important to ensure that the results are replicated in subsequent studies. However, most studies suggest that the post-legalization period was not associated with an increase in cannabis use for youth below the legal age, as it is the case for adolescents.^60^

### Clinical Implications

The present findings carry several clinical implications. First, the results highlight the importance of interventions that both target at-risk youth (e.g., those with high ADHD symptoms) and address contextual, environmental and mental health factors associated with later cannabis use. Specifically, clinicians managing adolescents with ADHD should not only consider these youths potentially at risk of early-onset use but should consider cannabis use as a behaviour that might exacerbate ADHD symptoms. This is especially critical given current studies suggesting that pharmacological treatment alone might not be sufficient to address the risk of substance use in youth with ADHD.^30^ In accordance with the multimodal approach of ADHD management, it would be important that effective addiction prevention interventions be offered to youth with ADHD. One type of program that could be highly relevant uses a personality-targeted approach, which allows for the personalization of drug prevention to an adolescent's trait vulnerability. A personality-targeted intervention has been shown to be effective in preventing adolescent substance use, and cannabis use specifically,^61,62^ while also reducing the risk of conduct disorder in youth at risk due to the impulsivity trait.^63^ It would therefore be important to confirm to what extent the effectiveness of this type of program is generalized to youth with ADHD, considering the high overlap between ADHD and impulsivity.

### Future Directions

Taken together, the present study highlights a bidirectional association between cannabis use and ADHD symptoms, especially between Grade 8 and Grade 10. Future studies clarifying the specific mechanisms underlying the prospective associations between cannabis use and ADHD symptoms would be highly valuable to inform targeted interventions. Based on prior research, it is plausible that cannabis contributes to subsequent exacerbation of ADHD symptoms via disruptions in executive functioning, which has itself been conceptualized as an etiological factor in ADHD. Further studies could also explore the contribution of other substances, such as tobacco, or further explore the moderating effect of sex and gender. Future studies should also aim to investigate the potential benefits of an intervention on the relationship between ADHD symptoms and cannabis use, particularly among youths with ADHD.

## Supplemental Material

sj-docx-1-cpa-10.1177_07067437261497468 - Supplemental material for Longitudinal Associations Between ADHD Symptoms and Cannabis Use in Adolescence: Exploration of the Dynamic Cross-Lagged Effects Supplemental material, sj-docx-1-cpa-10.1177_07067437261497468 for Longitudinal Associations Between ADHD Symptoms and Cannabis Use in Adolescence: Exploration of the Dynamic Cross-Lagged Effects by Julie Girard-Lapointe, Natalie Castellanos-Ryan, Samantha J. Lynch, Jeremy J. Watts, Maria Didaskalou and Patricia J. Conrod in The Canadian Journal of Psychiatry
