# Supplementary material for: Longitudinal Associations Between ADHD Symptoms and Cannabis Use in Adolescence: Exploration of the Dynamic Cross-Lagged Effects
Source: Can J Psychiatry. 2026 Nov 7:07067437261497468. Online ahead of print. doi: 10.1177/07067437261497468 (PMC13744488; doi:10.1177/07067437261497468)
Supplement: Supplementary file 1 [file sj-docx-1-cpa-10.1177_07067437261497468.docx]

Table S1. Path coefficients for the sex-stratified RI-CLPM

|  |  | T1 | | T2 | | T3 | | T4 | | T5 | |
| --- | --- | --- | --- | --- | --- | --- | --- | --- | --- | --- | --- |
| **Girls** | | β | *SE* | β | *SE* | β | *SE* | β | *SE* | β | *SE* |
|  | **Random intercepts** |  |  |  |  |  |  |  |  |  |  |
|  | RI by ADHD_t_ | 0.685***** | 0.015 | 0.692***** | 0.014 | 0.666***** | 0.015 | 0.672***** | 0.017 | 0.675***** | 0.013 |
|  | RI by Cannabis_t_ | 0.964***** | 0.027 | 0.499***** | 0.065 | 0.354***** | 0.052 | 0.334***** | 0.054 | 0.304***** | 0.050 |
|  | RI by Alcohol_t_ | 0.573***** | 0.060 | 0.475***** | 0.054 | 0.408***** | 0.046 | 0.381***** | 0.041 | 0.371***** | 0.046 |
|  | **Within factor** |  |  |  |  |  |  |  |  |  |  |
|  | ADHD | 0.728***** | 0.014 | 0.722***** | 0.013 | 0.746***** | 0.013 | 0.740***** | 0.016 | 0.737***** | 0.012 |
|  | Cannabis | 0.267** | 0.096 | 0.867***** | 0.037 | 0.935***** | 0.020 | 0.942***** | 0.019 | 0.953***** | 0.016 |
|  | Alcohol | 0.820***** | 0.042 | 0.880***** | 0.029 | 0.913***** | 0.021 | 0.925***** | 0.017 | 0.929***** | 0.019 |
|  | **Covariates** |  |  |  |  |  |  |  |  |  |  |
|  | Age → RI ADHD | 0.018 | 0.031 |  |  |  |  |  |  |  |  |
|  | Age → RI cannabis | 0.259***** | 0.064 |  |  |  |  |  |  |  |  |
|  | Age → RI alcohol | 0.259***** | 0.054 |  |  |  |  |  |  |  |  |
|  | SES → RI ADHD | -0.020 | 0.030 |  |  |  |  |  |  |  |  |
|  | SES → RI cannabis | 0.000 | 0.033 |  |  |  |  |  |  |  |  |
|  | SES → RI alcohol | 0.203***** | 0.046 |  |  |  |  |  |  |  |  |
|  | RI ADHD ↔ RI cannabis | 0.155***** | 0.051 |  |  |  |  |  |  |  |  |
|  | RI ADHD ↔ RI alcohol | 0.083 | 0.063 |  |  |  |  |  |  |  |  |
|  | RI cannabis ↔ RI alcohol | 0.547***** | 0.078 |  |  |  |  |  |  |  |  |
|  | **Within-person effects** |  |  |  |  |  |  |  |  |  |  |
|  | ADHD_t_ → ADHD_t_₊₁ | 0.236** | 0.076 | 0.351***** | 0.042 | 0.435***** | 0.034 | 0.047***** | 0.029 |  |  |
|  | Cannabis_t_ → Cannabis_t_₊₁ | -0.020 | 0.227 | 0.456***** | 0.046 | 0.523***** | 0.063 | 0.592***** | 0.045 |  |  |
|  | Alcohol_t_ → Alcohol_t_₊₁ | 0.359***** | 0.098 | 0.497***** | 0.045 | 0.508***** | 0.031 | 0.526***** | 0.035 |  |  |
|  | ADHD_t_ → Cannabis_t_₊₁ | 0.111***** | 0.034 | 0.060* | 0.031 | 0.030 | 0.036 | 0.043 | 0.029 |  |  |
|  | ADHD_t_ → Alcohol_t_₊₁ | 0.160* | 0.081 | 0.093** | 0.035 | 0.067** | 0.026 | 0.047 | 0.029 |  |  |
|  | Cannabisₜ → ADHDₜ₊₁ | -0.287 | 0.179 | 0.062***^+^*** | 0.032 | 0.068* | 0.034 | 0.050 | 0.033 |  |  |
|  | Cannabis_t_ → Alcohol_t_₊₁ | -0.339 | 0.210 | 0.042 | 0.029 | 0.120***** | 0.038 | 0.121***** | 0.033 |  |  |
|  | Alcohol_t_ → ADHD_t_₊₁ | 0.115 | 0.083 | 0.140** | 0.044 | 0.032 | 0.042 | 0.020 | 0.034 |  |  |
|  | Alcohol_t_ → Cannabis_t_₊₁ | 0.204***** | 0.063 | 0.204***** | 0.028 | 0.237***** | 0.054 | 0.152***** | 0.036 |  |  |
|  | **Residual covariances** |  |  |  |  |  |  |  |  |  |  |
|  | ADHD_t_ ↔ Cannabis_t_ | -0.006 | 0.219 | 0.109 | 0.078 | 0.154***** | 0.029 | 0.055* | 0.027 | 0.122***** | 0.032 |
|  | ADHD_t_ ↔ Alcohol_t_ | 0.262***** | 0.034 | 0.095 | 0.117 | 0.136***** | 0.045 | -0.004 | 0.035 | 0.060* | 0.027 |
|  | Cannabis_t_ ↔ Alcohol_t_ | 0.025 | 0.213 | 0.214***^+^*** | 0.112 | 0.350***** | 0.028 | 0.265***** | 0.033 | 0.269***** | 0.045 |
| **Boys** | |  |  |  |  |  |  |  |  |  |  |
|  | **Random intercepts** |  |  |  |  |  |  |  |  |  |  |
|  | RI by ADHD_t_ | 0.704***** | 0.018 | 0.694***** | 0.021 | 0.699***** | 0.020 | 0.693***** | 0.019 | 0.694***** | 0.022 |
|  | RI by Cannabis_t_ | 0.848***** | 0.066 | 0.752***** | 0.051 | 0.518***** | 0.071 | 0.429***** | 0.056 | 0.371***** | 0.051 |
|  | RI by Alcohol_t_ | 0.702***** | 0.040 | 0.645***** | 0.041 | 0.566***** | 0.035 | 0.481***** | 0.030 | 0.457***** | 0.032 |
|  | **Within factor** |  |  |  |  |  |  |  |  |  |  |
|  | ADHD | 0.710***** | 0.018 | 0.720***** | 0.020 | 0.715***** | 0.020 | 0.721***** | 0.018 | 0.720***** | 0.021 |
|  | Cannabis | 0.530***** | 0.105 | 0.659***** | 0.058 | 0.855***** | 0.043 | 0.903***** | 0.027 | 0.929***** | 0.020 |
|  | Alcohol | 0.713***** | 0.039 | 0.764***** | 0.034 | 0.825***** | 0.024 | 0.877***** | 0.017 | 0.889***** | 0.016 |
|  | **Covariates** |  |  |  |  |  |  |  |  |  |  |
|  | Age → RI ADHD | 0.066* | 0.027 |  |  |  |  |  |  |  |  |
|  | Age → RI cannabis | 0.252* | 0.056 |  |  |  |  |  |  |  |  |
|  | Age → RI alcohol | 0.237***** | 0.062 |  |  |  |  |  |  |  |  |
|  | SES → RI ADHD | 0.034 | 0.028 |  |  |  |  |  |  |  |  |
|  | SES → RI cannabis | 0.016 | 0.035 |  |  |  |  |  |  |  |  |
|  | SES → RI alcohol | 0.208***** | 0.037 |  |  |  |  |  |  |  |  |
|  | RI ADHD → RI cannabis | 0.230***** | 0.037 |  |  |  |  |  |  |  |  |
|  | RI ADHD → RI alcohol | 0.143***** | 0.041 |  |  |  |  |  |  |  |  |
|  | RI cannabis → RI alcohol | 0.581***** | 0.077 |  |  |  |  |  |  |  |  |
|  | **Within-person effects** |  |  |  |  |  |  |  |  |  |  |
|  | ADHD_t_ → ADHD_t_₊₁ | 0.234***** | 0.030 | 0.263***** | 0.053 | 0.315***** | 0.044 | 0.404***** | 0.037 |  |  |
|  | Cannabis_t_ → Cannabis_t_₊₁ | -0.184 | 0.240 | 0.362***** | 0.043 | 0.493***** | 0.044 | 0.575***** | 0.039 |  |  |
|  | Alcohol_t_ → Alcohol_t_₊₁ | 0.129 | 0.094 | 0.353***** | 0.051 | 0.451***** | 0.030 | 0.498***** | 0.029 |  |  |
|  | ADHD_t_ → Cannabis_t_₊₁ | 0.036 | 0.068 | 0.090* | 0.037 | 0.109***** | 0.031 | 0.054***^+^*** | 0.031 |  |  |
|  | ADHD_t_ → Alcohol_t_₊₁ | 0.149***** | 0.034 | 0.060* | 0.028 | 0.075* | 0.035 | 0.000 | 0.043 |  |  |
|  | Cannabisₜ → ADHDₜ₊₁ | -0.069 | 0.076 | 0.080* | 0.035 | 0.075* | 0.030 | 0.035 | 0.034 |  |  |
|  | Cannabis_t_ → Alcohol_t_₊₁ | -0.120 | 0.141 | 0.071* | 0.036 | 0.111**** | 0.039 | 0.096* | 0.041 |  |  |
|  | Alcohol_t_ → ADHD_t_₊₁ | 0.053 | 0.046 | 0.096 | 0.030 | -0.020 | 0.035 | 0.052* | 0.024 |  |  |
|  | Alcohol_t_ → Cannabis_t_₊₁ | -0.099 | 0.107 | 0.119* | 0.047 | 0.144***** | 0.030 | 0.202***** | 0.027 |  |  |
|  | **Residual covariances** |  |  |  |  |  |  |  |  |  |  |
|  | ADHD_t_ ↔ Cannabis_t_ | -0.003 | 0.080 | 0.092 | 0.067 | 0.117***** | 0.035 | 0.098** | 0.035 | 0.061***^+^*** | 0.034 |
|  | ADHD_t_ ↔ Alcohol_t_ | 0.081* | 0.041 | 0.134* | 0.037 | 0.083** | 0.029 | 0.088* | 0.039 | 0.058***^+^*** | 0.032 |
|  | Cannabis_t_ ↔ Alcohol_t_ | -0.092 | 0.148 | 0.080 | 0.108 | 0.323***** | 0.039 | 0.296***** | 0.030 | 0.306***** | 0.029 |

*SE = Standard Error;* ***^+^****=p-value ≤0.10; *=p-value ≤0.05; **=p-value≤0.01; ***=p-value≤0.001;*

Model fits. Boys: χ²(74)= 197.974, *p*=.00, CFI=.987, TLI= .977, RMSEA= .030, 90%Cl [.025, .035], SRMR = .041. Girls: χ²(73)= 193.097, *p*=.00, CFI=.986, TLI= .975, RMSEA= .030, 90%Cl [.025, .035], SRMR = .034.

Table S2. Model fit indices

|  | CFI | TLI | RMSEA [90%Cl] | SRMR | χ²(df), *p* |
| --- | --- | --- | --- | --- | --- |
| Model 1 | .988 | .979 | .028 [.024, .031] | .034 | 336.695 (87), 00 |
| Model 2 | .982 | .968 | .027 [.024, .030] | .034 | 324.671 (87), 00 |
| Model 3: Boys | .987 | .977 | .030 [.025, .035] | .041 | 197.974 (74), 00 |
| Model 3: Girls | .986 | .975 | .030 [.025, .035] | .034 | 193.097 (73), 00 |

CFI=Comparative Fit Index, TLI=Tucker-Lewis Index, RMSEA=Root Mean Square Error of Approximation, CI=Confidence Interval, SRMR=**Standardized Root Mean Squared Residual**

Table S3. Indirect effect of significant cross-lagged paths

|  | β | *SE* | Lower CI (95%) | Higher CI (95%) |
| --- | --- | --- | --- | --- |
| ADHD T2 → Cannabis T3 → ADHD T4 | 0.006***^+^*** | 0.003 | 0.002 | 0.014 |
| ADHD T3 → Cannabis T4 → ADHD T5 | 0.003 | 0.002 | 0.001 | 0.009 |
| Cannabis T2 → ADHD T3 → Cannabis T4 | 0.006* | 0.003 | 0.002 | 0.014 |
| Cannabis T3 → ADHD T4 → Cannabis T5 | 0.003 | 0.002 | 0.000 | 0.009 |
| ADHD T2 → Cannabis T3 → ADHD T4 → Cannabis T5 | 0.000 | 0.000 | 0.000 | 0.001 |
| Cannabis T2 → ADHD T3 → Cannabis T4 → ADHD T5 | 0.000 | 0.000 | 0.000 | 0.001 |

CI=Confidence Interval; *SE = Standard Error;* ***^+^***=p-value ≤0.10; *=p-value ≤0.05
